# Hepatitis C virus drives the pathogenesis of hepatocellular carcinoma: from immune evasion to carcinogenesis

**DOI:** 10.1038/cti.2016.55

**Published:** 2016-10-07

**Authors:** Miriam Canavese, Danushka Wijesundara, Guy J Maddern, Branka Grubor-Bauk, Ehud Hauben

**Affiliations:** 1 Liver Metastasis Research Group, Discipline of Surgery, The Basil Hetzel Institute for Translational Health Research, The Queen Elizabeth Hospital, University of Adelaide, Adelaide, South Australia, Australia; 2 Virology Laboratory, Discipline of Surgery, The Basil Hetzel Institute for Translational Health Research, The Queen Elizabeth Hospital, University of Adelaide, Adelaide, South Australia, Australia

## Abstract

Persistent hepatitis C virus (HCV) infection is associated with high incidence of hepatocellular carcinoma (HCC), the most common primary malignancy of the liver with over half a million new cases diagnosed annually worldwide. The aryl hydrocarbon receptor (AhR) is a ubiquitously expressed transcription factor and its activation by environmental chemicals and by its endogenous ligand kynurenine (Kyn) has been implicated in a variety of tumour-promoting processes such as transformation, tumorigenesis and in immunosuppression that enables tumour survival and growth. Kyn is generated constitutively by human tumour cells via tryptophan (Trp)-2,3-dioxygenase (TDO), a Trp-degrading enzyme expressed in liver, brain and cancer cells. Notably, it has been shown that TDO-derived Kyn suppresses anti-tumour immune responses, thus promoting tumour-cell survival through activation of the AhR pathway. In the context of HCV infection-associated HCC, it was shown that AhR signalling is increased in HCV-infected hepatocytes, and that modifications in the expression of AhR pathway-specific genes are associated with the progression of HCV infection into HCC. Based on these observations, we present and discuss here the hypothesis that HCV infection promotes HCC by modulation of the TDO–Kyn–AhR pathway, resulting in tumorigenesis as well as in suppression of both anti-HCV and anti-tumour immune responses.

## Presentation of the hypothesis

We propose herein that hepatitis C virus (HCV) infection promotes hepatocellular carcinoma (HCC) by augmentation of the tryptophan (Trp)-2,3-dioxygenase-kynurenine-aryl hydrocarbon receptor (TDO–Kyn–AhR) pathway, resulting in suppression of both anti-HCV and anti-tumour immune responses, as well as in tumorigenesis.

The evolution of HCV into HCC was associated with expression of specific AhR pathway genes. Although only few genes were found to be differentially expressed in HCV-induced HCC tumour biopsies compared with paired non-HCC liver sections, pathway analysis revealed strong upregulation of genes involved in AhR signalling in biopsies from HCV-induced HCC tumours.^1, 2, 3^ In addition, it has been shown that dioxin-induced persistent AhR activation promotes tumour formation, carcinogenicity and clonal expansion of transformed cells by inhibiting apoptosis and bypassing AhR-mediated cell cycle arrest.^4^ Notably, it has been recently shown that an endogenous ligand promotes the activation of AhR under physiological conditions without the presence of exogenous toxic chemicals.^5^ As described by Tian *et al.*,^6^ AhR is a highly evolutionary conserved, ligand-activated transcription factor that is best known to mediate the toxicities of dioxins and dioxin-like compounds. The non-activated AhR resides in the cytoplasm and, after binding its ligand, translocates to a nucleus where it reacts with its obligatory heterodimer partner AhR nuclear translocator, to become fully competent and bind the promoter element of specific target genes.^6^ Functionally, AhR was shown to have an important role in the development and differentiation of lymphocytes.^7^

Opitz *et al.*^5^ have identified the Trp catabolite Kyn as an endogenous ligand of human AhR. Interestingly, these authors also showed that TDO-derived Kyn was able to inhibit anti-tumour effects and promote tumour-cell survival and dissemination through a direct effect on the AhR pathway. They also demonstrated the active presence of the TDO–Kyn–AhR pathway in human brain tumours, which correlated with disease progression and poor survival. This novel association between Trp derivatives and the AhR pathway creates new links among a large body of scientific literature regarding the following: (a) the immune suppressive effects of indoleamine-2,3-dioxygenase (IDO), a therapeutic target being evaluated in a number of ongoing clinical trials (reviewed in Vachelli *et al.,*^8^ and Munn and Mellor^9^). Interestingly, whereas the level and activity of TDO in HCV or HCC has not been previously investigated, upregulation of hepatic IDO expression and an increased serum Kyn:Trp ratio was demonstrated in patients with chronic HCV infection and in infected chimpanzees.^10, 11^ Moreover, hepatic IDO expression decreased in animals that cleared the infection, but remained high in those that progressed to chronicity;^10^ (b) the immunosuppressive effects of TDO;^12, 13^ and (c) the immunosuppressive and tumorigenic effects of the AhR pathway. Based on the above evidence, we propose that manipulation of the TDO–Kyn–AhR pathway by HCV has a pathogenic role in the HCV-infected liver through bystander suppression of anti-tumour immune responses, thus allowing the progression of HCV infection into HCC. Accordingly, we suggest that identification of biological or chemical compounds that inhibit the TDO–Kyn–AhR pathway can lead to the development of new therapeutic strategies for HCV-induced HCC.

## Context of the hypothesis

The Trp to Kyn metabolic pathway is a key regulator of innate and adaptive immunity.^14, 15^ It has been shown that Trp metabolism is targeted by viruses to escape immune control in a number of chronic viral infections including HCV, hepatitis B virus (HBV), human immunodeficiency virus, herpes and cytomegalovirus (reviewed in Mehraj and Routy^16^). Moreover, a plethora of research evidence suggests that this immune tolerance pathway, driven by the key, rate-limiting enzymes IDO in many tissues and by TDO in the liver, also has a regulatory role in cancer immunity, autoimmunity, transplant rejection and allergy.^12, 15^ In cancer patients, IDO expression was demonstrated in various types of tumours including prostate, colorectal and pancreatic cancer,^17^ and IDO1 expression was associated with poor prognosis in ovarian cancer patients.^18^ Based on these findings, drugs targeting the IDO pathway are being tested in clinical trials,^8^ with the aim of preventing tumour-induced immunosuppression. However, recent studies^5, 13^ demonstrating that TDO-mediated Trp metabolism represents an alternative route to IDO activity employed by tumour cells generated growing interest in TDO as therapeutic target for cancer immunotherapy in general and, in particular, liver cancers.^19^ Thus, as the available IDO inhibitors do not suppress TDO activity, there could be a need to develop specific TDO inhibitors.^15^

An extensive research effort was dedicated to characterize the regulatory mechanisms of Trp metabolism and of the downstream pathways mediating its effects on immunological functions, including the AhR pathway.^15^ Trp catabolites were shown to have an important balancing role in both antimicrobial defence and immune regulation, at least in part through Kyn-mediated AhR activation.^16^ Therefore, IDO/TDO-mediated AhR activation generates conflicting effects by (a) depleting L-Trp to starve the invading pathogens and (b) contributing to downregulation of the inflammatory response against microorganisms that survived the acute response.^16^ IDO-mediated degradation results in Trp deprivation, affecting various pathogens including certain bacteria, parasites and occasionally viruses.^16^ Nevertheless, chronic viral infections result from manipulation of the host immune response via Kyn-mediated AhR activation to create a state of immune tolerance.^16^ It is known that various malignancies occur in the context of chronic infection and inflammation,^20^ where local immune suppression is sustained by Trp catabolism in the tumour microenvironment.^21^ Importantly, Opitz *et al.*^5^ implicated Kyn as the endogenous tumour-promoting ligand of the AhR in various cancers. Therefore, these authors showed that activation of the IDO/TDO–Kyn–AhR pathway in response to inflammatory stimuli constitutes a novel link between inflammation and carcinogenesis.

**Figure 1:**
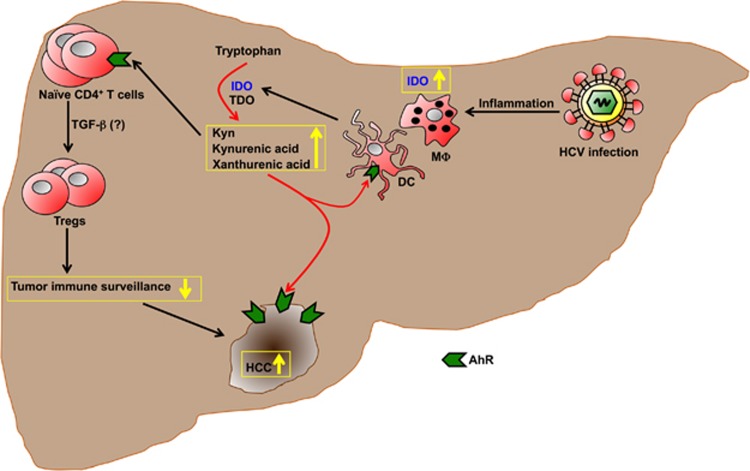
IDO/TDO-based HCC development and progression following persistent HCV infection. Persistent HCV infection leads to chronic inflammation, which recruits immune cells expressing IDO such as dendritic cells (DCs) and macrophages (MΦ) to the liver. This increases the levels of IDO in the liver, which along with TDO participates in the catabolism of Trp into metabolites such as Kyn, kynurenic acid and xanthurenic acid that can activate AhR signalling. Elevated levels of AhR ligands, in particular Kyn, engage with AhR on naive CD4^+^ T cells present in the liver. AhR signalling possibly coupled with signalling from cytokines such as transforming growth factor (TGF)-β leads to differentiation of naive CD4^+^ T cells to Tregs that can suppress immune responses that can prevent formation of HCC. We hypothesize that this immune suppression pathway leads to the establishment of HCC. HCC also express AhR, which following activation contributes to HCC progression. Furthermore, DC also express AhR and activation of this receptor on DC is known to increase IDO expression, which can also contribute to immune suppression using the illustrated pathway. The yellow arrows indicate up/downregulation of metabolites or a process. Furthermore, red arrows indicate catabolic enzymatic reaction of Trp, which leads to elevated AhR signalling, and the black arrows are now exclusively present to indicate the direction of the flow of the diagram.

HCC is the most prevalent primary malignancy of the liver; it is the fifth most common cancer in men and seventh among women, with over 500 000 new cases diagnosed worldwide each year;^22, 23, 24^ yet, effective therapeutic options for advanced HCC are limited. Therefore, characterization of the mechanisms mediating the progression to HCC in HCV-infected patients is a worldwide medical priority. Cirrhosis due to chronic HBV or HCV infection is the most critical pathogenic factor for HCC.^25^ Indeed, global epidemiology of HCC is associated with the prevalence of viral hepatitis and the age it is acquired in. HCV is an RNA virus, which does not integrate its genome into the DNA of host cells. In most cases, failure to clear the virus results in chronic infection associated with progressive risk of hepatic fibrosis, cirrhosis and HCC.^26^ The DNA damage and oxidative stress induced by chronic hepatic inflammation are the major correlated causes of HCV-associated HCC.^27, 28, 29^ The large body of research evidence supporting the direct role of HCV in promotion of liver cancer is summarized in Figure 1.^30, 31, 32, 33^

In chronic liver disease, altered gene expression can be mediated by epigenetic changes, by inhibition of DNA repair and/or by differential expression of microRNAs. These alterations include constitutive upregulated expression of ‘stemness' factors, suggesting that both HCV and HBV contribute to HCC by promoting stemness,^34^ referring to the capability to self-renew and differentiate into other tumour cell types.^35^ Notably, HCV- and HBV-encoded proteins regulate cancer-associated host gene expression patterns and cellular phenotypes. These changes can result in growth factor-independent proliferation, drug resistance, tissue invasion and metastasis.^36^ Chronic inflammation can also promote somatic mutations in tumour cells. The contribution of HCV to HCC involves the core protein and non-structural proteins NS3 and NS5A, which have been shown to contribute to oncogenic transformation.^34, 37, 38^ Changes in host gene expression that promote tumorigenesis also seem to support virus replication and/or protect virus-infected hepatocytes from immune-mediated damage or destruction. Therefore, tumour-promoting changes in local immunity seem to simultaneously sustain chronic viral infection and promote HCC carcinogenesis. Thus, the pathogenesis of HCC is closely correlated with virus persistence in infected cells over a long period of time.^34^ Nevertheless, HCV persistence does not include DNA integration and the virus maintains itself as an endoplasmic reticulum-associated episome. Therefore, progressive chronic liver disease is associated with increased proportion of virus-infected liver cells that acquire certain characteristic of malignant cells.^34, 39^ As a result, persistent infections can accelerate the pathogenesis of HCC through common necro-inflammatory pathways or by direct oncogenic activity.^40^

Infection-induced inflammation triggers catabolism of Trp via IDO/AhR.^16^ In human, Trp catabolism through the Kyn pathway is regulated by three distinct enzymes: IDO-1, IDO-2, which are inducible in many tissues, and TDO, which is expressed in liver, brain and in cancer cells.^41, 42^ In physiological conditions, TDO is the main enzyme degrading Trp (reviewed in Platten *et al.*^15^ and Mehraj and Routy^16^), while in the context of infections: IDO-1 is induced and becomes the major intracellular enzyme mediating Trp degradation. Thus, in chronic viral infections IDO induction is considered the main cause of decreased serum Trp levels.^16^ It has been shown that both natural and induced human T regulatory (Treg) cells have an important role in the progression of HCV infection to HCC and are associated with the severity of viral recurrence after liver graft transplantation.^43, 44^ Although nothing is known with regards to the specific impact of HCV on these two Treg cell populations, Ouaguia *et al.*^45^ have suggested that HCV promotes recruitment and induction of Treg cells in the infected liver, mediating their phenotype and suppressive activity to induce immune tolerance and allow the progression of liver disease. This suggestion was based on the findings showing that HCV infection (a) induces Treg cell-mediated anergy, (b) promotes the recruitment of Treg cells by HCV-infected hepatocytes and (c) induces significant increase in the expression of Treg markers, such as CD25 and FOXP3, thus potentiating Treg suppressive function. Moreover, the authors demonstrated that HCV promotes conversion of naive T lymphocytes into induced Treg type 1 (Tr1) cells, which could act as another mechanism for HCV to induce immune tolerance towards viral antigens. Notably, the fact that HCV can potentiate the suppressive function of natural Treg cells as well as induce Tr1 cells may help to explain the cellular mechanism by which HCV escapes the immune system to promote the progression of hepatitis C infection to cirrhosis and HCC.^45^ However, the molecular mechanisms mediating HCV immune evasion, potentially including IDO/TDO, Kyn and AhR activation, remain to be further characterized.

It has been previously demonstrated that AhR activation induces antigen-specific active tolerance through direct and dendritic cell-mediated effects on Treg cell survival and function.^46^ In addition, more recent work done by Mascanfroni *et al.*^47^ reported that a metabolic programme controlled by the transcription factor hypoxia-inducible factor-1α (HIF-1α) and by AhR promotes the differentiation of Tr1 cells. Notably, this work showed that the balance between HIF-1α and AhR provides a pathway for modulating the immune response in response to immunological, metabolic and environmental signals. Thus, AhR induces the degradation of HIF-1α and subsequently regulates the differentiation of Tr1 cells from naive T cells. On the other hand, extracellular ATP and hypoxia, linked to inflammation, trigger AhR inactivation by HIF-1α and inhibit Tr1 cell differentiation. Therefore, the cross-talk between HIF-1α and AhR provides a system for immune modulation based on the bioenergetic needs of specific cell subsets.^47^ Moreover, dimerization of HIF-1β or AhR nuclear translocator with HIF-1α is involved in various aspects of carcinogenesis, including proliferation and survival under hypoxic conditions: to this end, Choi *et al.*^48^ have recently demonstrated suppression of tumour cell invasion and migration in HIF-1β-silenced HCC cell lines, suggesting that HIF-1β expression is essential for tumour cell survival under hypoxic conditions in HCC. The competition between tissue resident cells and circulating immune cells on the limited locally available resources can result in ‘natural selection' of immune phenotypes at the site of inflammation.^49^

Therefore, through its interactions with both immune and metabolic systems, HCV activates multiple direct and indirect pathogenic pathways, including liver fibrogenic pathways, cellular and survival pathways.^50^

Cozzi *et al.*^51^ showed that patients with chronic HCV infection have lower serum Trp concentrations compared with healthy donors. IDO, the rate-limiting enzyme of the Kyn pathway, produces several metabolites, which are also AhR ligands.^52^ Kyn is one such catabolite that regulates immune functions by acting as an AhR agonist, at least in part by promoting the induction and activation of immunosuppressive Treg cells.^53, 54^ Indeed, it has been shown that IDO1-expressing dendritic cells exert broad and robust immunosuppressive effects such as directly suppressing the proliferation and effector functions of cytotoxic T lymphocytes, NK cells and plasma cells,^8, 55, 56^ promoting the induction of CD4^+^CD25^+^FOXP3^+^ Treg cells from naive CD4^+^ T cells^57, 58^ and triggering immunosuppressive activity in neighbouring IDO1-expressing dendritic cells.^57, 59^ Notably, the upregulation of IDO1 in plasmocytoid dendritic cells^60^ has been shown to contribute to the immunosuppressive activity of human immunodeficiency virus-1.^61, 62^ Taken together, these findings reinforce the idea that IDO/TDO–Kyn–AhR pathway mediates robust immunosuppressive effects in both physiological and pathological contexts.

The direct and indirect links between the AhR pathway and HCC tumorigenesis were further outlined by functional characterization of the AhR nuclear translocator, also known as HIF-1β, which is widely expressed in human cells, including hepatocytes.^48^ When responding to different extracellular stimuli, AhR nuclear translocator can form a heterodimeric complex with AhR, HIF-1α and its homologous factors (HIF-2α and HIF-3α), to mediate various biological actions such as hypoxia reaction,^47^ xenobiotic metabolism and immunosuppression,^1, 63^ other than being an important regulator of HCC growth and metastasis, and therefore a promising prognostic candidate in HCC patients.^1^ In line with our hypothesis, preclinical studies have suggested that AhR is responsible for mediating, at least in part, the immunosuppressive effects of cancer-derived Trp metabolites^5, 54^ and, therefore, represents a logical pharmaceutical target for cancer immunotherapy.^15^ Specifically, in the context of HCV-associated HCC, it was shown that AhR signalling is impaired in HCV-infected hepatocytes, and that changes in mRNA expression of specific genes in the AhR pathway are linked to progression of HCV infection to HCC.^2, 3^ Briefly, the Kyn pathway is regulated by IDO1, IDO2 and TDO.^41, 42^ These three enzymes actively deplete Trp from the circulation, thus producing several catabolites, known as Kyns.^64^ Some of these metabolites such as kynurenic acid and quinolinic acid regulate neuronal functions,^65^ whereas the amino acid L-Kyn has immunosuppressive effects, through the AhR and other pathways as well. Notably, the immunomodulatory AhR ligand L-Kyn is the first stable Trp catabolite in this pathway, whereas some of its downstream derivatives including 3-hydroxykynurenine, anthranilic, kynurenic, quinaldic, xanthurenic and 3-hydroxyanthranilic have been shown to have a role in neurodegenerative disorders.^65^Therefore, based on the above findings we propose herein that HCV infection promotes HCC tumour survival and growth by activating theTDO–Kyn–AhR pathway, resulting in the following: (a) tumorigenesis, (b) suppression of the anti-HCV immune response and (c) bystander suppression of anti-tumour immune responses. Moreover, the proposed hypothesis supports the development of therapeutic interventions specifically targeting the TDO–Kyn–AhR pathway in HCV-infected patients.

## Testing the hypothesis

Based on this hypothesis, we propose that the TDO–Kyn–AhR pathway can be characterized in blood samples from patients with acute HCV infections, persistent HCV infections and no HCC, persistent HCV-associated HCC and healthy controls. The aim of discovery efforts could be to identify AhR pathway-related targets and predictive biomarkers representing molecular checkpoints in the progression of HCV infection into HCC. In the second phase, the capacity of biological/chemical AhR pathway modulators to regulate the expression and/or function of relevant AhR pathway targets will be evaluated, with the aim of developing novel therapeutic strategies for HCV and HCV-induced HCC. This approach could reveal molecular mechanisms involved in HCV-persistent infection and progression into HCC, and thus facilitate the development of prognostic surveillance approach in HCV patients for early detection of HCC, valuable research tools such as predictive *in vitro* assays and AhR antagonist compounds, as well as new therapeutic avenues.

## Implications of the hypothesis

In summary, the identification and characterization of the link among TDO, Kyn and AhR, including its negative feedback mechanisms,^66^ may pave the way for targeted therapeutic interventions to allow abrogation of HCV immune evasion mechanisms and bystander suppression of anti-HCC immune responses. New directions include further examination into development and clinical testing of Trp immune-metabolic pathway inhibitors, AhR pathway inhibitors, as well as the possibility of combination therapy with non-redundant immune checkpoint inhibitors, such as those targeting the programmed death-1, T-cell immunoglobulin mucin receptor 3 and cytotoxic T-lymphocyte-associated protein 4 pathways.^8, 67^ Such immunological approach in patients with chronic viral infections using immune checkpoint inhibitors and/or interleukin-7 may result in different safety profiles as compared with similar interventions in cancer patients.^68, 69, 70, 71^ Marra *et al.*^72^ characterized the induction of HCC by viral factors and identified disease biomarkers of HCC pathogenesis. The development of HCC in HCV-infected patients requires up to 30 years from primary infection.^73^ However, the course of HBV-related carcinogenesis is less predictable, as in some patients HCC can even precede cirrhosis, in particular with chronic HBV infection in endemic areas.^74^

In conclusion, the most effective tool for HCC prevention is avoiding risk factors such as viral infection. An effective vaccine has been available for prevention of new infection with HBV; however, to date, no vaccine against HCV infection has been approved.^72^

Importantly, the changes in signalling pathways and gene expression, which are induced by viral proteins in hepatocytes, are often mutated in HCC.^75^ Therefore, virally triggered epigenetic modification of tumour suppressor genes can allow the constitutive expression of oncogenes in early tumorigenesis and mutation in these same oncogenes result in higher constitutive expression that supports tumour survival and growth.^76^ Thus, inflammation-induced oncogene expression is an early event in HCC.^34^ As malignant cell clones expand, they acquire heritable epigenetic changes that result in a permanent change in phenotype.^76^ Molecular characterization of these changes will be a fertile ground for the identification of candidate biomarkers and targets for therapeutic intervention. Moreover, future investigations of Trp metabolism and its links with the AhR pathway will be instrumental for the development of therapeutic approaches to break the active immune tolerance towards viral antigens, cure chronic viral infections and prevent hepatic conditions such as cirrhosis and cancer.
